# Chimeric Antigen Receptor T-Cell Therapy in Metastatic Castrate-Resistant Prostate Cancer

**DOI:** 10.3390/cancers14030503

**Published:** 2022-01-20

**Authors:** Mahasha P.J. Perera, Patrick B. Thomas, Gail P. Risbridger, Renea Taylor, Arun Azad, Michael S. Hofman, Elizabeth D. Williams, Ian Vela

**Affiliations:** 1 School of Biomedical Sciences at Translational Research Institute (TRI), Queensland University of Technology (QUT), Brisbane, QLD 4102, Australia; pb.thomas@qut.edu.au (P.B.T.); ed.williams@qut.edu.au (E.D.W.); 2 Queensland Bladder Cancer Initiative (QBCI), Woolloongabba, QLD 4102, Australia; 3 Department of Urology, Princess Alexandra Hospital, Brisbane, QLD 4102, Australia; 4 Centre for Personalised Analysis of Cancers (CPAC), Brisbane, QLD 4102, Australia; 5 Prostate Cancer Research Group, Monash Biomedicine Discovery Institute Cancer Program, Department of Anatomy and Developmental Biology, Monash University, Clayton, VIC 3168, Australia; gail.risbridger@monash.edu (G.P.R.); renea.taylor@monash.edu (R.T.); 6 Prostate Cancer Theranostics and Imaging Centre of Excellence (ProsTIC), Cancer Imaging, Peter MacCallum Cancer Centre, Melbourne, VIC 3000, Australia; arun.azad@petermac.org (A.A.); michael.hofman@petermac.org (M.S.H.)

**Keywords:** prostate cancer, chimeric antigen receptor therapy, CAR-T, metastatic castrate-resistant prostate cancer, adoptive cell transfer, adoptive immunotherapy

## Abstract

**Simple Summary:**

Prostate cancer is one of the most frequently diagnosed cancers amongst men worldwide. Treatment for metastatic disease is often in the form of androgen deprivation therapy. However, over the course of treatment affected men may become castrate-resistant. Options for men with metastatic castrate-resistant cancer are limited. This review focuses on the role of chimeric antigen receptor T-cell therapy (CAR-T) in men with metastatic castrate-resistant prostate cancer. This review is a contemporary appraisal of preclinical and clinical studies conducted in this emerging form of immunotherapy. A thorough evaluation of the role of CAR-T therapy in prostate cancer is provided, as well as the obstacles we must overcome to clinically translate this therapy for men affected with this rapidly fatal disease.

**Abstract:**

Prostate cancer is the most commonly diagnosed solid-organ cancer amongst males worldwide. Metastatic castrate-resistant prostate cancer (mCRPC) is a rapidly fatal end-sequelae of prostate cancer. Therapeutic options for men with mCRPC are limited and are not curative in nature. The recent development of chimeric antigen receptor T-cell (CAR-T) therapy has revolutionised the treatment of treatment-resistant haematological malignancies, and several studies are underway investigating the utility of this technology in the treatment of solid tumours. In this review, we evaluate the current treatment options for men with mCRPC as well as the current landscape of preclinical and clinical trials of CAR-T cell therapy against prostate cancer. We also appraise the various prostate cancer-specific tumour-associated antigens that may be targeted by CAR-T cell technology. Finally, we examine the potential translational barriers of CAR-T cell therapy in solid tumours. Despite preclinical success, preliminary clinical trials in men with prostate cancer have had limited efficacy. Therefore, further clinically translatable preclinical models are required to enhance the understanding of the role of this investigational therapeutic in men with mCRPC. In the era of precision medicine, tailored immunotherapy administered to men in a tumour-agnostic approach provides hope to a group of men who otherwise have few treatment options available.

## 1. Current Treatment Options

Prostate cancer is the most frequently diagnosed solid-organ cancer amongst men, and the second-leading cause of cancer-related death in men worldwide [1]. Localised cases of prostate cancer can be managed with surgery or radiation therapy; however, approximately 20% of patients diagnosed with prostate cancer will develop metastatic disease [2]. This can initially be managed with androgen deprivation therapy (ADT); however, over time virtually all patients will progress to develop castrate-resistant prostate cancer (CRPC). Time to progression from hormone-sensitive prostate cancer (HSPC) to castrate resistance occurs at a median of 16.5 months [3]. The prognosis of men with castrate-resistant prostate cancer (CRPC) is poor. Patients may develop CRPC prior to metastatic disease; however, recent studies suggest up to 84% of patients with CRPC have concurrent metastatic disease [4]. Men with metastatic CRPC (mCRPC) have a median survival of 10 months to 21.7 months and a five-year survival rate of 30% [5]. To date, there are no curative treatments available for mCRPC, with current treatment approaches centred on improving progression-free survival (PFS), overall survival (OS) and quality of life (QoL).

Docetaxel was the first effective therapeutic agent demonstrated to prolong survival in men with mCRPC, in two large-scale phase III trials in 2004 [6,7]. The development of novel anti-androgen agents and androgen synthesis inhibitors has significantly changed the treatment paradigm for men with prostate cancer. Several large-scale multicentre trials have led to improvement in progression-free and overall survival in men with metastatic HSPC using abiraterone acetate (STAMPEDE and LATITUDE) [8,9], enzalutamide (ARCHES and ENZAMET) [10,11] and apalutamide (TITAN) [12]. Enzalutamide has recently been demonstrated to provide improved OS and PFS with mCRPC, compared to placebo in the pre- (PREVAIL) [13] and post-chemotherapy (AFFIRM) [14] settings. Abiraterone has also demonstrated similar results in chemotherapy-treated (COU-AA301) [15] and chemotherapy-naïve settings (COU-AA302) [16]. In fact, recent trials suggest both enzalutamide (PROSPER) [17] and apalutamide (SPARTAN) [18] have improved overall survival in the nonmetastatic CRPC setting.

Despite the prolonged PFS and OS achieved by anti-androgenic agents, approximately 20–40% of patients have primary resistance to these agents, and virtually all patients will develop secondary resistance [19]. In patients who have disease progression after docetaxel therapy and androgen-signalling targeted inhibitors, cabazitaxel, a next-generation taxane, has recently emerged as an option after demonstrating improved median radiological PFS, PFS and OS [20]. The median overall survival for patients treated with cabazitacel versus anti-androgen agents was 12.6 months versus 11 months; however, 53% of patients experienced grade 3 or 4 adverse events. Whilst these treatments prolong overall survival, they are not a cure for the disease and treatments have temporary benefit, with significant adverse effects. This highlights the need for tailored treatments in men with mCRPC. In the last few years, several therapeutic agents have targeted men with mCRPC. Currently, approved therapies from the Food and Drug Administration (FDA) include sipuleucel-T, radium-223, poly (ADP-ribose) polymerase (PARP) inhibitors and checkpoint inhibitors.

Radiopharmaceutical agents allow for the delivery of targeted radiotherapy to sites of metastases. Radium-223 is an alpha-emitter that selectively binds to areas of high bone turnover and emits high-energy alpha-particles. A phase 3 trial involving 921 patients demonstrated a survival benefit of 3.6 months versus the placebo in men with mCRPC [21]. Given 90% of men with CRPC have skeletal metastatic disease [22], radium-223 provides a potential treatment, particularly for those men with symptomatic bony metastatic disease. More recently, the prostate-specific membrane antigen (PSMA) directed radioisotope lutetium-177 ([^177^Lu] Lu-PSMA-617) has emerged as another promising targeted therapy. The TheraP trial, a multicentre unblinded phase 2 trial, recruited 200 men with mCRPC to cabazitaxel versus [^177^Lu] Lu-PSMA-617 treatment. The [^177^Lu] Lu-PSMA-617 arm had a higher percentage of men with a prostate-specific antigen (PSA) decline of >50% and fewer Grade 3–4 adverse events compared to the cabazitaxel arm [23]. The VISION trial was an international open-label phase 3 trial of [^177^Lu] Lu-PSMA-617 treatment in men with mCRPC. Sartor and colleagues were able to demonstrate prolonged imaging-based progression-free survival and overall survival across 831 mCRPC patients treated with of [^177^Lu] Lu-PSMA-617 and standard care compared to standard care alone [24]. Several other trials are underway investigating [^177^Lu] Lu-PSMA-617 in combination with PARP inhibitors in the mCRPC setting [25], as well as in combination with chemotherapy (such as docetaxel) in the HSPC setting [26].

Recently, the PROfound [27] and TRITON2 [28] trials have highlighted the clinical utility of PARP inhibitors such as olaparib and rucaparib, in patients with specific gene alterations in DNA repair pathways. This was following the improved objective response rates demonstrated in individuals harbouring homologous recombination repair (HRR) defects such as BRCA1/BRCA2/ATM mutations [27,28]. The role of combinatorial therapy with PARP inhibitors, anti-androgen agents and immune checkpoint inhibitors is unknown; they are currently under clinical investigation [29,30]. Whilst prostate cancer has traditionally been recognised as an immunologically ‘cold’ disease, several immunotherapeutic agents have been somewhat successful in men with mCRPC. In 2011, sipuleucel-T, the first vaccine-based therapy, was approved by the FDA for use in men with symptomatic mCRPC. This followed a double-blinded multicentre phase 3 trial of 512 patients that demonstrated a 4.1-month improvement in median survival in the sipuleucel-T group compared to the placebo group [31]. Early trials investigating immune checkpoint inhibitors trials such as ipilimumab in men with mCRPC have not been remarkable [32,33]. The KEYNOTE-199 study demonstrated an objective response rate of only 5% by a cohort of men receiving pembrolizumab monotherapy with proven tumour PD-1 expression [31]. Despite this, pembrolizumab has been approved based on precise tumour-agnostic features. Patients possessing mismatch repair-deficient (dMMR) tumours or cyclin-dependent kinase 12 (CDK12) loss appear to respond well to checkpoint inhibition [34,35,36]. Advances in the genomic sequencing of clinical material and the success of agents for tumour-specific features have highlighted the need for precision therapies targeting a patient’s individual tumour profile.

Recently, adoptive cellular immunotherapies (ACT) have been developed. ACT involves the transfer of immunogenic cells into the patient to elicit an antitumourigenic response. Modalities include chimeric antigen receptor T cell (CAR-T) therapy, tumour-infiltrating lymphocytes (TILs), T-cell receptor therapy (TCR) and bispecific engagers (BiTEs). CAR-T cell therapy involves the generation of autologous genetically engineered T-cells, which target and immunologically attack specific tumour-associated antigens (TAAs). CAR-T cell therapy has transformed the treatment of treatment resistant or refractory haematological malignancies in the last few years.

The first clinical application of CAR-T cell therapy was investigated in young adults with B-cell acute lymphoblastic leukaemia (B-ALL). A phase I trial of 53 young adults undergoing anti-CD19-directed CAR-T cell therapy in adults with relapsed B-ALL demonstrated complete remission in 83% of patients at a median follow-up at 29 months [37]. This led to the FDA approving the first CAR-T cell therapy, tisagenlecleucel. A month after the FDA approval, axicabtagene ciloleucel was approved for the treatment of refractory large B-cell lymphoma [38]. In July of 2020, a third CAR-T cell treatment, brexucabtagene autoleucel, was approved for adults with chemoresistant mantle cell lymphoma [39]. The success of CAR-T cell therapy has prompted investigation into a variety of solid tumours, including prostate cancer.

**Figure 1:**
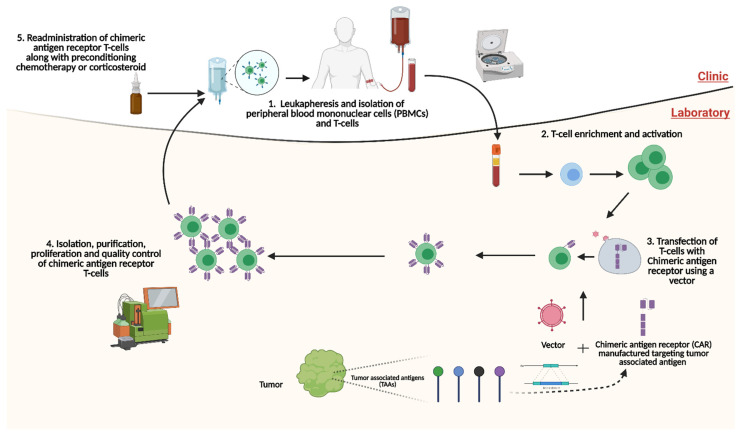
Platform of autologous administration of chimeric antigen receptor T-cell (CAR-T) therapy. Through leukapheresis, T-cells are isolated, activated and enriched. CARs targeting TAAs are synthesised and transfected into T-cells through a vector. Following this, CAR-T cells are isolated, purified and proliferated. CAR-T cells are then readministered to the patient, typically after the patient has had preconditioning treatment with either a steroid or chemotherapy. CAR: chimeric antigen receptor; PBMCs: peripheral blood mononuclear cells; TAAs: Tumour-associated antigen. Created with BioRender.com (accessed on 1 December 2021).

The platform of CAR-T cell administration is autologous and relies on the ex vivo development and enrichment of T-cells (Figure 1). The process begins with the leukapheresis of the patient’s blood to isolate peripheral blood mononuclear cells (PBMCs). T-cells are isolated and transfected with the CAR protein, which has been genetically engineered to target the TAA. Transfection of T-cells can be performed using a retroviral, lentiviral or nonviral vector [40]. Once transfection is a completed, molecular expansion of the T-cell population is undertaken and this population is purified ex vivo. The purified T-cells expressing the CAR protein, now termed CAR-T cells, are then administered back to the patient. Typically, before the administration of CAR-T cells, the patient undergoes lymphodepletion via chemotherapy or corticosteroids. Lymphodepletion serves multiple purposes in the context of CAR-T cell therapy, namely (1) increased persistence, expansion, and function of the CAR-T cells; (2) nullification of endogenous T-regulatory cells and other immunosuppressive cells; and (3) greater availability of pro-inflammatory cytokines through the elimination of their peripheral utilisation or ‘haematopoietic sinks’ [41]. Several preclinical and clinical trials are underway investigating CAR-T cell therapies within the context of prostate cancer. This review will provide a comprehensive overview of CAR-T cell therapy in mCRPC as well as the challenges of this translational immunotherapeutic modality.

## 2. Chimeric-Antigen Receptor T-Cell (CAR-T Cell) Structure

**Figure 2:**
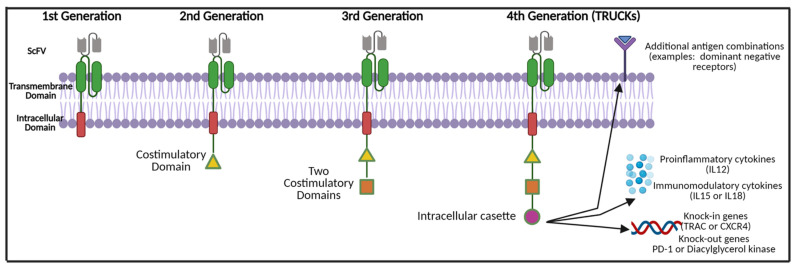
CAR-T cell modifications and evolution throughout generations. Abbreviations: ScFV—single chain variable fragment; IL—interleukin; TRUCKS: T-cells redirected for universal cytokine mediated killing; TRAC—T-cell receptor α constant, CXCR4—chemokine receptor type 4; PD-1—programmed death cell protein 1. Created with BioRender.com (accessed on 1 December 2021).

The structure of a CAR-T cell is comprised of three main components: (1) an extracellular domain, (2) a transmembrane domain, and (3) the intracellular zone (Figure 2). The extracellular domain is composed of a single-chain fragment variable (scFV) that is designed to bind to a TAA [42]. The scFV is linked to the T-cell itself via the transmembrane domain. The transmembrane domain is composed of proteins (examples: FcεRI, CD3, CD8 and CD28) that are attached to an intracellular domain [42,43]. The intracellular zone harbours the immune receptor tyrosine-based activation motif (ITAM), which plays a central role in signal transduction for T-cell activation. Initial in vivo testing of the fundamental first-generational CAR-T cell structure demonstrated poor T-cell activation and persistence [44]. Therefore, several additional molecules were added to the intracellular domains as this therapy was further developed.

Second-generation CAR-T cells possess a second intracellular costimulatory protein such as CD28, CD27, CD134 or CDB7 [45,46]. Additional T-cell activation was achieved by incorporating two costimulatory proteins in the intracellular zone (such as the addition of 4-1BB or CD 3ζ), thus producing the third generation of CAR-T cells [47,48]. The latest fourth-generation CAR-T cells, also known as ‘TRUCKs’ (T-cells redirected for universal cytokine mediated killing), are armed with a variety of motifs within the intracellular cassette. These may include pro-inflammatory cytokine inducers of interleukin (IL)-12, which assist in combating the immunosuppressive tumour microenvironment and shift T-cells to T-helper-1-type cells [49]. Instead of proinflammatory cytokines, TRUCKs may possess inducers of IL-15 that enhance T-memory stem cells or IL-18. This aims to counteract cytokine toxicity [50]. Other non-cytokine-based molecules have been added to the TRUCK cassette to either enhance the effectiveness of therapy or prevent excessive toxicity. These include knock-out genes (such as PD-1 or diacylglycerol kinase) and knock-in genes (such as TRAC or CXCR4) [51,52]. Additional controlled and inducible systems (Syn/Notch), antigen combinations (HER2+ IL-13R alpha 2) have been synthesised that aim to prevent antigen escape [51,53,54].

The key strength of CAR-T cell therapy is that specific tumour antigens can be targeted to direct an immunological response, independent of antigen-presenting cells (APCs), as well as the major histocompatibility complex system (MHC) [55]. As there is no reliance on the MHC system for antigen presentation and processing, CAR-T cells are insensitive to tumour escape mechanisms commonly mediated by the MHC. Such mechanisms recognised in prostate cancer include the downregulation of MHC Class I expression, or manipulation of the tumour microenvironment such that there are fewer tumour–MHC Class I epitope interactions [56]. Additionally, CARs can target antigens such as glycolipids, glycosylated proteins and conformational epitopes that are not readily recognised by T-cell receptors [57]. As such, administration of CAR-T cells to patients has the beneficial effects of bypassing immunological tolerance and enhanced tumour antigen targeting. The most vital initial step in CAR-T cell therapy development is the identification of an appropriate TAA.

## 3. Current Prostate Cancer Tumour-Associated Antigen (TAAs)

TAAs possess an epitope that is specific to cancerous tissue, and weakly expressed or absent on healthy tissue. It does not necessarily need to be target tissue-specific (i.e., prostate-specific), if the TAA is ubiquitously expressed in cancerous cells. Furthermore, the TAA should lack heterogeneity in expression across tumour cells and be able to constitutively direct an immunological response effecting cell death [58]. With respect to CAR-T therapy, TAAs should also be expressed on the cellular surface. Several TAAs have been investigated within prostate cancer and are outlined in Table 1.

### 3.1. Prostate-Specific Antigen (PSA)

Given serum PSA is frequently used as a surrogate marker of prostate cancer disease control in patients, preclinical studies have explored PSA as a target. Arredouani et al. induced PSA-specific cytotoxic lymphocytes after the immunization of humanised and castrated hybrid mice with a PSA-expressing recombinant vaccinia virus [59]. However, a significant limitation of PSA as a chimeric target is the fact that a predominant hypotype of free-PSA is expressed in benign tissue in men [71]. Therefore, as the prostate cancer microenvironment possess several immunosuppressive elements, PSA-targeted therapies may preferentially target healthy tissue.

### 3.2. Prostate Acid Phosphatase (PAP)

PAP is a tyrosine phosphatase protein secreted by both malignant and benign prostate cells [72]. It is also expressed in other organs such as the kidneys, testes, and bladder [52,60]. PAP has been successful as an immuno-oncological target, as evidenced by sipuleucel-T [31]. However, this TAA also has several limitations. PAP is more highly expressed in lower Gleason 6 and 7 tumours than in higher-grade tumours [52]. PAP is also exponentially secreted into the blood in large amounts when prostate tissue is damaged, which may contribute to off-tumour target toxicity [73].

To date, the most extensively investigated TAAs preclinically and clinically in the context of prostate cancer are prostate stem cell antigen (PSCA), epithelial cell adhesion molecule (EpCAM) and prostate-specific membrane antigen (PSMA).

### 3.3. Prostate Stem Cell Antigen (PSCA)

PSCA is a cell membrane glycoprotein that is expressed by prostate cells. Expression rates of PSCA in prostate cancer tissue are almost 90% higher than in benign tissue [74]. Additionally, a positive correlation exists with PSCA expression and advanced clinical disease. Gu and colleagues examined 120 primary prostate cancer and metastatic specimens and found that the level of PSCA expression was associated with higher Gleason score, higher tumour stage and androgen independence [74]. Furthermore, 100% of the metastatic specimens examined demonstrated expression of PSCA. PSCA is also exclusively expressed on the cell surface and not released into the blood [73]. Hence, the biological features of PSCA are favourable for immunological targeting. PSCA has been investigated as a potential target for antibody-based immunotherapy.

Morgenroth et al. first developed anti-PSCA CAR-T cells in 2007 and were able to demonstrate effective lysis of PSCA-expressing cells [75]. Since then, several preclinical models have been evaluated with PSCA-specific CAR-T cells. A limiting factor of in vitro testing is that there are no prostate (or other) cancer cell lines that endogenously and uniformly express PSCA culture conditions. As such, these cell lines are either transfected or transduced to express PSCA. PSCA-CAR-T cells have demonstrated specific and effective cytokine release as well as cell lysis in vitro against a variety of cell lines that have been genetically transduced to express PSCA (RT4, LAPC-9, DU145 and PC-3) [75,76,77,78,79,80,81].

These results have been recapitulated in vivo in immunocompromised mice using a variety of prostate cancer models (PC-3, PC-3M, mel526 and LAPC-9). CAR-T-treated mice displayed tumour regression and prolonged survival compared to mice bearing untreated tumour xenografts [76,77,78,79,80,81]. Whilst the majority of xenograft models displayed almost complete tumour volume reduction, in some cases tumour growth kinetics were only partially reduced in CAR-T cell-treated xenografts [77]. Nevertheless, this is impressive given the preclinical murine models were not preconditioned prior to CAR-T cell therapy. A few preclinical studies have, however, examined tumour-dependent CAR-T cell trafficking. Of note, Priceman et al. examined tumourigenic effects in a biologically relevant orthotopic intratibial tumour model. Intravenously administered PSCA-CAR-T cells showed near complete regression of the intratibial tumours and effective T-cell trafficking via noninvasive optical imaging [79]. Priceman et al. also compared CAR-T cells containing CD28 versus 4-1BB costimulatory domains and found that the latter were more effective in activating T-cells. Nevertheless, given the immunosuppressive prostate cancer tumour microenvironment, further studies are warranted examining trafficking to sites of bony metastases, particularly in syngeneic xenograft models.

In addition to inadequate T-cell trafficking, another mechanism of CAR-T cell treatment failure is the upregulation of PD-1/PD-L pathway in the tumour microenvironment. Increased binding of PD-1/PD-L receptors between T-cells and the tumour leads to immunosuppression of T-cells and, thus, less effective immune-mediated tumour cytotoxicity [81]. Recently, Zhou et al. utilised short-hair pin RNA technology (shRNA) to generate PD-1 silenced PSCA-specific CAR-T cells. They were able to demonstrate superior in vivo and in vitro results against two subcutaneous tumour models compared to non-PD-1 silenced CAR-T cells [81]. In addition to PD-1 silencing, dual-targeting CAR-T cells have been created to mitigate antigenic escape. Feldmann and Kloss et al. created co-expressing anti-PSCA and anti-PSMA CAR-T cells with in vivo results superior to their uni-targeting CAR-T counterparts [76,78]. Hence, PSCA-targeted CAR-T therapies have demonstrated clear promise preclinically. Given PSCA expression is upregulated on prostatic bony metastatic disease, this TAA may be particularly relevant for mCRPC. However, further bony metastatic models are required preclinically to assess effective CAR-T cell trafficking to these sites.

The impressive preclinical findings have resulted in the development of early phase I and phase II clinical trials investigating PSCA CAR-T cells, which are still in progress. To date, there is one clinical trial of PSCA-directed treatment within mCRPC specifically. The City of Hope Medical Center (Duarte, CA, USA) has a phase I trial underway on three patient cohorts based on the presence of preconditioning (fludarabine and cyclophosphamide) and escalating doses of CAR-T cells [82]. As PSCA is expressed in gastric and pancreatic tumours, two other open-label trials are in progress [83,84]. Another U.S. multicentre phase I trial is investigating PSCA-specific CAR-T cells (‘BPX-601′- Bellicum Pharmaceuticals(Houston, TX, USA) in combination with, rimiducid, a protein-dimerizing agent that purports to stabilise CAR-T cells and facilitate expansion and survival in vivo [83]. This study is enrolling patients with PSCA-positive gastric, pancreatic and prostate cancers. Patients recruited with prostate cancer will have mCRPC. These trials are likely to provide important clinical data on CAR-T therapy in the mCRPC setting. A summary of all preclinical and clinical trials using PSCA-specific CAR-T therapy is provided in Table 2.

### 3.4. Epithelial Cell Adhesion Molecule (EpCAM)

EpCAM, also known as CD236, is a transmembrane glycoprotein expressed in several solid tumours, including prostate cancer. EpCAM is expressed on a subset of normal epithelia but highly overexpressed in malignant cells and cancerous stem cells of a variety of solid cancers [85]. EpCAM expression plays a crucial role in the metastatic progression of tumours by preventing cell–cell adhesion and facilitating cell migration, proliferation and differentiation. It is therefore not prostate-specific, but is ubiquitously overexpressed in solid tumours. Hence, EpCAM is being investigated in a variety of solid tumours such as breast, gastric, colorectal, ovarian and nasopharyngeal carcinomas, as well as intraperitoneal carcinomatosis [64].

Ni and colleagues utilised shRNA to knock down EpCAM expression in prostate cancer cell line PC-3 xenografts [86]. They subsequently treated these xenografts with either radiotherapy or chemotherapy and demonstrated increased sensitivity to chemotherapy and radiation in the EpCAM knocked-down mice. Interestingly, they were able to concomitantly highlight, through immunohistochemistry, that EpCAM-knockdown is associated with downregulation of the PI3K/Akt/mTOR pathway. The PI3K/Akt/mTOR pathway is a well-recognised pathway of castrate resistance [86]. This is particularly pertinent clinically, as EpCAM-directed therapies, alongside conventional chemoradiation, may result in enhanced antitumourigenic effects.

Deng et al. studied EpCAM CAR T-cells against PC-3 and PC-3M (a metastatic clone of the PC-3) [87]. Firstly, they demonstrated that PC-3M cells had higher levels of EpCAM expression than PC-3 cell lines. Furthermore, EpCAM CAR-T cells produced significant antitumourigenic results both in vivo and in vitro against both the PC-3 and PC-3M human prostate cancer cells. Following injection of PC-3 tumours to the mouse tail vein, lung and bone metastases were noted. In the EpCAM-treated group, all mice survived at the 80-day mark and showed significant regression of both lung and bone metastases. This is in contrast to the control group of untreated mice, in which one-third of the mice survived at the 80-day mark.

A disadvantage of EpCAM directed therapy is dose-dependent off-tumour on-target pulmonary toxicity secondary to alveolar EpCAM expression [65]. There is an overall paucity of preclinical studies specifically examining the effect of EpCAM adoptive treatment on prostate cancer cells, so further data are required. Clinically, there has only been one open-label clinical trial in patients with EpCAM expressing prostate, colon, oesophageal, pancreatic or hepatic cancer. Whilst the trial was due for completion in 2020, the current status of the trial is unknown [88]. A current summary of preclinical and clinical studies on EpCAM-targeted therapies is provided in Table 2.

**Table 2:** Current preclinical and clinical trials involving prostate stem cell antigen (PSCA) and epithelial cell adhesion molecule (EpCAM).

Prostate Stem Cell Antigen (PSCA)						
Completed Preclinical Studies						
Author	Location	Intervention		Model		
Morgenroth et al. 2007 [75]	University of Cologne, Germany	PSCA-specific CAR-T cells		HEK cell line 293^(PSCA +)^		
Kloss et al. 2013 [76]	Memorial Sloan Kettering Cancer Centre, New York	Combinatorial low-affinity PSCA CAR-T cells + high-affinity PSMA CAR-T Cells		CD19^(PSMA+)^, PC-3 ^(PSCA+)^, PC-3^(PSMA+PSCA+)^ cell lines PC-3^(PSMA+/−PSCA+/−)^ xenografts		
Hillerdal et al. 2014 [77]	Uppsala University, Sweden	3rd generation PSCA-specific CAR-T cells		mel526 ^(PSCA +)^ and mel526 ^(TARP+)^ cell lines mel526 xenografts		
Feldmann et al. 2017 [78]	Institute of radio-pharmaceutical cancer research, Germany	2nd Generation PSCA and PSMA ‘UniCAR-T’ cells		PC-3^(PSMA+ PSCA+)^ and LNCaP-C4-2B^PSCA+^ cell lines PC-3^(PSMA+ PSCA+)^ xenografts		
Priceman et al. 2018 [79]	City of Hope Medical Centre, California	2nd Generation PSCA- (CD28) or (4-1BB)—CAR-T cells		PC-3^(PSCA+)^, DU-145^(PSCA+)^ cell lines LAPC-9^PSCA+^ xenografts		
Han et al. 2020 [80]	Hebei Medical University, Shijiazhuang, China	3rd Generation minicircle DNA-PSCA-CAR T cells		RT4^(PSCA+)^ PC-3M^(PSCA+)^ cell lines PC-3-M^(PSCA+)^ xenografts		
Zhou et al. 2021 [81]	East China Normal University, China	3rd Generation PSCA/PD-1 silencing CAR-T cells		PC3^(PSCA+)^ PC3^(PSCA+)^ xenografts		
**Ongoing Clinical Trials**						
**Identifier**	**Location**	**Type**	**Intervention**	**Primary Endpoints**	**Enrolment**	**Completion Date**
NCT03873805 [82]	City of Hope Medical Centre, California United States	Phase I	PSCA-specific CAR-T cells + cyclophosphamide +fludarabine Subjects with PSCA positive mCRPC	Safety and tolerability	33	February 2021
NCT02744287 [83]	Multiple centres, United States	Phase I	PSCA-specific CAR-T cells + Rimiducid (dimerization agent) Subjects with previously treated advanced tumour (Pancreatic + prostate cancer)	Safety and dose limiting toxicity	151	February 2024
NCT03198052 [84]	Second Affiliated Hospital of Guangzhou Medical University, China	Phase I	CAR-T cells targeting HER2, Mesothelin, PSCA, MUC1, Lewis-Y, GPC3, AXL, EGFR, Claudin18.2, or B7-H3 Patients with advanced cancer	Safety and dose limiting toxicity	30	August 2023
**Epithelial cell adhesion molecule (EpCAM)**						
**Completed Preclinical Studies**						
**Author**	**Location**	**Intervention**		**Model**		
Deng et al. 2015 [87]	Cancer Hospital and Institute, China	EpCAM-specific CAR-T cells		PC-3 and PC-3M cell line PC-3 xenografts		
**Ongoing Clinical Trials**						
**Identifier**	**Location**	**Type**	**Intervention**	**Primary Endpoints**	**Enrolment**	**Type**
NCT03013712 [88]	First Affiliated Hospital of Chengdu Medical College, China	Phase I/II	EpCAM-specific CAR-T cells EpCAM positive prostate, colon, oesophageal, pancreatic, hepatic carcinomas.	Safety and dose limiting toxicity	60	December 2020 (unknown status)

Brackets denote the cell line was transduced to express PSCA or PSMA.

### 3.5. Prostate-Specific Membrane Antigen (PSMA)

PSMA has been the most extensively investigated prostate cancer TAA to date. PSMA is a type II transmembrane protein expressed on the prostate epithelial membrane. PSMA, as a target antigen, is advantageous in that it is expressed in all grades of prostate cancer and progressively increases in expression in higher-grade tumours [66]. Furthermore, the expression of PSMA is upregulated with the emergence of castration resistance and de-differentiation [66,70]. PSMA is also expressed in other tissues of the body, namely the small bowel, kidneys, salivary glands and ovarian tissue—however, at a lower concentration than in prostate or seminal fluid. PSMA is also universally upregulated in the neovasculature of several tumour entities. This highlights the potential role of PSMA in the angiogenesis of tumours and their associated metastatic processes [68]. PSMA has already played a central role in diagnostic imaging and theranostics. PSMA Positron Emission Tomography/Computed Tomography (PET/CT) molecular imaging has revolutionised prostate cancer imaging in the last five years. PSMA PET/CT uses a small molecule that binds to the PSMA receptor on prostate cancer cells linked to a nuclear medicine imaging isotope (gallium-68). This imaging modality has clearly been demonstrated to be superior to conventional medical imaging modalities [89]. Thus, PSMA CAR-T cell treatments have an added advantage in that treatment can be easily imaged in humans compared to other TAAs.

Many in vivo and in vitro studies examining PSMA-directed CAR-T therapy have demonstrated impressive results. CAR-T therapies have been tested on multiple prostate cancer cell lines that either endogenously express PSMA, such as (LNCaP, C4-2B), or against cell lines transduced to express PSMA (PC-3, DU145). Maher et al. first examined PSMA-directed T-cells in vitro and demonstrated cytotoxic-specific lysis of PSMA-expressing prostate cancer cell lines [90].

Gade and colleagues subsequently examined CAR-T cell therapy in three different prostate cancer xenograft models [91]. They demonstrated that 55% of treated xenografts had complete responses and long-term survival compared to control groups, in orthotopic, subcutaneous and in lung metastatic models. Subsequent immunohistochemical analysis showed unchanged expression of PSMA in both untreated and treated tissues, thus excluding antigen downregulation/loss (antigen escape) as a cause for partial responses. A similar result was also demonstrated by Zuccolotto et al., with all treated mice demonstrating longer survival and 60% of mice displaying complete eradication of bioluminescent tumours in a pulmonary metastasis model [92]. To date, no bony metastasis models have been evaluated for anti-PSMA CAR-T cell therapy. This is a significant limitation as 90% of metastatic deposits in prostate cancer are within the bone [22].

Whilst it is clear, preclinically, that targeted CAR-T cells have antitumourigenic responses, very few studies have examined combinatorial treatment with clinically relevant treatments such as chemotherapy or anti-androgens. A study by Alzubi et al. is the only study to date that has investigated such combination therapies preclinically [93]. They highlighted that systemic infusion of CAR-T cells, in combination with non-ablative low-dose docetaxel, significantly inhibited tumour growth in murine models compared to CAR-T cell treatment alone. Interestingly, whilst focal injections of CAR-T cells alone completely eradicated tumours, Alzubi and colleagues demonstrated that monotherapeutic administration of CAR-T cells or docetaxel were not effective in systemic therapy. As men with mCRPC are usually on an androgen blockade and sometimes chemotherapy, further studies combining CAR-T cell therapy are warranted. Other preclinical studies have examined variation in the structure of PSMA-directed CAR-T cells. The addition of two costimulatory domains (CD28 and 4-1BB) together led to superior effects compared with the use of one costimulatory domain alone [93,94,95]. This notion, however, was recently challenged by another study performed by Zuccolotto et al. [96], which showed that CD28-harbouring anti-PSMA CAR-T cells were more effective than combined CD28 and 4-1BB anti-PSMA CAR-T cells. Despite the contention regarding improved efficacy between second- and third-generation CAR-T cells, second^-^generation CAR-T cells are unequivocally shown to be superior to first-generation CAR-T cells. Ma et al. established a γ-irradiation animal model and highlighted the role of nonmyeloablative preconditioning in CAR-T treatment and the overall superiority of second-generation CAR-T cells [97].

It also appears that fourth-generation CAR-T cells are clearly superior in efficacy compared to third-generation CAR T-cells. Recently, fourth-generation CAR-T cells have been designed with additional moieties to mediate the cytokine response. Wang et al. examined anti-IL23, anti-PSMA CAR-T cells against prostate cancer cell lines and a xenograft model [98]. IL-23 is produced by myeloid-derived suppressor cells (MDSCs) that can activate the androgen receptor pathway, promoting cell survival [99]. Wang and colleagues demonstrated enhanced tumour eradication in the anti-IL23 targeted group of mice versus standard PSMA CAR-T-treated mice.

All preclinical studies that have examined the effect of adding an anti-TGF-β moiety to the CAR-T construct have shown greater T-cell recruitment, cytokine release and cytotoxicity in vitro, as well as greater tumour-growth suppression in vivo [100,101,102]. It should be noted that, while Kloss et al. demonstrated complete eradication of some tumours in vivo, some mice developed xenogeneic graft versus host disease after PSMA and TGF-β targeted therapy. These toxicities may be overcome through the insertion of various ‘suicide genes’ that can cause T-cell death when activated by a drug. Zhang et al. added a herpes simplex virus thymidine kinase (HSV-tk) ‘suicide gene’ into their CAR-T cell construct, which inhibited CAR-T cells in vivo following ganciclovir administration [100]. However, this raises questions regarding its clinical tolerability within humans.

The first clinical trial of anti-PSMA CAR-T cells was suspended for financial reasons [103]. Junghans et al. administered first-generation PSMA CAR-T cells to five patients; two of the five patients achieved PSA declines of 50% and 70%, whereas the remaining patients had disease progression. Whilst these results were poor, the study utilised a first-generational CAR-T construct, which has clearly been shown to be inferior in T-cell recruitment and expansion. This was reflected as the study identified suboptimal levels of IL-2 within subjects, and a further phase II trial was planned with an optimised protocol. No off-tumour toxicities were observed in any of the patients.

Preliminary results of another ongoing phase I/II dose-escalation trial conducted by Slovin and colleagues investigated second-generation anti-PSMA CAR-T cell therapy [104]. The seven participants were divided into two cohorts based on dosing, four of whom received 1 × 10^9^ cells/kg and three of whom received 1.5–3 × 10^9^ cells/kg. Two patients in the first cohort had stable radiological disease for >6 months and >16 months, respectively, while the other patients had disease progression. The patients in the latter group all developed mild cytokine release syndrome that was self-resolving. The researchers also demonstrated that the CAR-T cells lasted for two weeks. The tumour response of the second cohort of patients is still under investigation.

Two particularly important clinical trials are investigating anti-PSMA CAR-T cells co-targeting TGF-β [105,106]. Both trials aim to build upon the observation that, in vivo, the addition of a dominant negative TGF-β receptor increases CAR-T cell efficacy [100,101,102]. A cohort of this trial will not be given the standard preconditioning cyclophosphamide, thus addressing the question of preconditioning in CAR-T therapy. Hence, the initial preliminary clinical trials of CAR-T cell therapy in men with prostate cancer has indicated tolerability, but with suboptimal efficacy. Additional clinical trials across larger cohorts of patients are required to further characterise the toxicity as well as the efficacy. All completed or ongoing trials are summarised in Table 3.

Unfortunately, approximately 10–15% of men with advanced prostate cancer have disease that expresses low or absent levels of PSMA [111]. This is particularly relevant for patients who possess ductal or neuroendocrine variants of prostate cancer, as there is increasing evidence that these cancers express lower levels of PSMA [69,70]. Hence, other novel prostate cancer-specific target TAAs have been identified and tested preclinically.

## 4. Novel TAAs under Investigation

Many novel prostate cancer TAAs have been investigated recently in relation to therapeutic targets. Newly emerging prostate cancer-specific TAAs are summarised in Table 4 below.

## 5. Challenges in Solid Tumours

**Figure 3:**
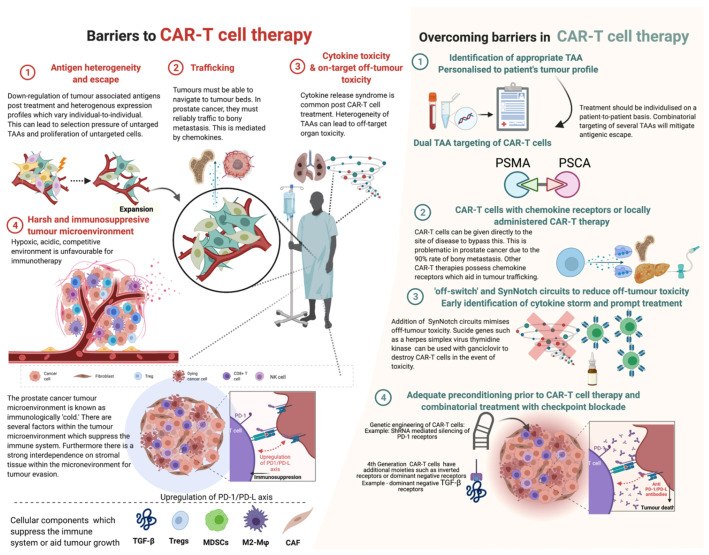
Barriers in CAR-T cell therapy and strategies adopted in overcoming these barriers. Abbreviations: TGF-β—tumour growth factor beta; Tregs—T regulatory cells; MDSCs—myeloid-derived stem cells; M2-Mφ—M2 macrophages; CAF—cancer-associated fibroblasts; PD-1—programmed cell death type 1; PD-L—programmed death ligand; shRNA—short hairpin RNA. Created with BioRender.com (accessed on 1 December 2021).

Whilst CAR-T cell therapy has revolutionised the treatment of haematological malignancies, this success has largely not been repeated in solid tumours. The reasons for this are thought to be due to many factors (Figure 3).

Unlike haematological malignancies, solid tumours express antigenic heterogeneity rather than monoclonality [125], and therefore some tumours in certain individuals may express a combination of antigens that may differ from another individual’s. Intratumoural heterogeneity is also common in prostate cancer; hence, an individual tumour itself may display a wide variety of antigens. Furthermore, there is evidence that CAR-T treatment can induce antigen escape by means of adaptive resistance [126]. Given the low tumour mutational burden and the antigenic heterogeneity, a useful approach is manufacturing CARs that target multiple antigens. Kloss et al. and Feldmann et al. created CARs that target PSCA and PSMA, demonstrating effective results [76,78].

CAR-T cells must be able to appropriately traffic to tumour bed sites. Prostate cancer-directed CAR-T cells must be able to display efficient trafficking, particularly to sites of skeletal metastatic disease. Trafficking itself is mediated by chemokines; however, T-cells lack cognate chemokine receptors and tumours frequently produce the small amounts of chemokines necessary for sufficient trafficking [125]. This can logically be bypassed by local administration of the CAR-T cells to the tumour bed directly. However, this strategy has not been extensively studied in prostate cancer due to the frequent presence of diffuse macro and micro-metastatic/oligometastatic skeletal disease. A strategy to overcome this is to add chemokine receptors on the T-cells themselves. Moon and colleagues attached chemokine receptors to mesothelin-targeted CAR-T cells for malignant pleural mesothelioma and demonstrated a 12.5-million-fold increase in T-cell tumour infiltration [127].

Once the CAR-T cells traffic to the appropriate sites, the T-cells should be able to navigate the immunosuppressive tumour microenvironment (TME). There is substantial evidence of the interaction between epithelial prostate cancer cells and the surrounding stromal cellular tissue. The prostate cancer TME provides a hypoxic, acidic and competitive environment that often impairs immunological cells from affecting tissue. Additionally, solid TMEs often demonstrate upregulation of immunosuppressive cytokines and molecules such as tumour-associated macrophages (TAM), myeloid-derived suppressor cell (MDSC) and T-regulatory cells (Tregs) [128]. O’Rourke et al. conducted immunohistochemistry on surgical specimens matched to pre- and post-CAR-T cell therapy and showed a significant upregulation of immunosuppressive molecules (such as indoleamine 2,3-dioxygenase (IDO), programmed death (PD) ligand 1 (PD-L1) and TGF-β [126].

This phenomenon could be potentially mitigated by co-administration of immune checkpoint inhibitors, PD-1 antibodies, or direct inhibition of other immunosuppressive molecules. To date, Kloss et al. and Zhang et al. have investigated CAR-T cells with dominant negative TGF-β receptor showing markedly improved results. A current clinical trial of anti-PSMA, dominant negative TGF-β CAR-T cells is underway. Co-administration of immune checkpoint inhibitors or PD-1/PD-L antibodies has not been evaluated in vitro or in vivo. A recently evaluated approach is to selectively knock-out or knock-in key endogenous genes through targeted nucleases using CRISPR-mediated genome editing [129].

Despite the abundance of well-designed approaches to overcome these barriers, a fundamental issue that remains is the selection of appropriate preclinical models. Pertinent preclinical models that directly relate to men with mCRPC are insufficient in the current literature. Skeletal and visceral metastatic models, as well as patient-derived xenografts, provide tumour heterogeneity and microenvironments that are more clinically relevant. Thus, further preclinical evaluation utilising these models are indispensable to our understanding of not only the efficacy of treatment but the mechanisms of failure of CAR-T cell therapy.

## 6. Adverse Effects

### 6.1. On-Target Off-Tumour Toxicity

On-target off-tumour toxicity refers to direct attack on normal tissues that share expression of the TAA [130]. Most prostate TAAs are also strongly expressed in benign tissue along with malignant tissue, hence raising the concern of on-target off-tumour toxicity. Early prostate cancer clinical trials and murine studies have not highlighted any off-tumour toxicities. However, as larger cohorts of patients are treated, this is always a potential risk, given the diverse range of tissues prostate TAAs are expressed in. This can be combated by targeting two antigens, as highlighted by Kloss et al. Furthermore, the addition of a synthetic notch receptor (SynNotch) as a strategy may minimise CAR-T cell toxicity [131]. SynNotch receptors are T-cell circuits, whereby the recognition of TAAs by a T-cell induces expression of a CAR toward a second antigen. Di Stasi and colleagues devised an inducible suicide gene as a ‘safety’ switch for CAR-T cell treatment [132]. When exposed to a synthetic dimerising drug, the inducible caspase 9 system becomes activated and leads to rapid death of the cells expressing this safety switch. Hence, further preclinical and clinical studies are required to specifically analyse off-tumour toxicities.

### 6.2. Cytokine Toxicity

A frequent side effect of CAR-T therapy is cytokine release syndrome (CRS). CRS is thought to be mediated by the release of various cytokines upon T-cell activation. These include IL-6, IFN-gamma, IL-8 and IL-10. CRS can occur 1–14 days after administration of treatment and is encountered in up to 80–100% of patients on CAR-T cell treatment [133]. The American Society for Blood and Bone Marrow Transplantation (ASBMT) consensus group published a grading score for CRS in which fever is a prerequisite for diagnosis, with hypotension and hypoxemia being the secondary determinants of the grading scale. Mild CRS can be managed with supportive treatment, and in more severe cases systemic corticosteroids can be administered. A short course of systemic steroids (<14 days) can rapidly reverse symptoms of CRS without compromising the anti-tumourigenic response [133]. Recently, the FDA has also approved tocilizumab, an IL-6 receptor monoclonal antibody [134]. Despite these treatment options, it is prudent to note that CRS can be fatal. Immune effector cell-associated neurotoxicity (ICAN) refers to the development of neurological symptoms secondary to cerebral oedema. Whilst its occurrence in CD19-directed treatment has been noted in up to 67% of patients, it has not yet been reported in solid tumours [135].

## 7. Conclusions

Whilst existing preclinical studies have shown promising results of CAR-T cell therapy against prostate cancer-associated tumour antigens, the translation to clinical trials has been disappointing. There are likely to be many reasons for this and our understanding of them is limited. Although clinical trials have been informative, the “failure” of many of these trials is ultimately detrimental to the enrolled patients. This highlights the critical need for biologically relevant preclinical models in order to improve the translation of CAR-T cell therapy into humans. The influence of intratumoural heterogeneity and the tumour microenvironment is more closely recapitulated in humanised xenograft models, but there is a paucity of these models in current prostate cancer collections. Further use of clinically translatable metastatic models, particularly skeletal metastatic xenograft models, will enable a better understanding of the true efficacy of this therapy, as well as the mechanisms that result in low efficacy of treatment. Only a small number of prostate cancer patients have been treated with CAR-T therapy to date, and data on the human off-tumour toxicities, optimal treatment combinations, durability, persistence and efficacy of treatment are mainly derived from studies in other tumour types. There is a necessity to establish the feasibility of treatment as the potential harms need to be balanced against the efficacy and durability of results. Men with mCRPC have a poor prognosis and a generally rapid demise, and there is a significant unmet need for tailored treatments in a tumour-agnostic approach. In the era of precision medicine, CAR-T cell therapy provides hope to patients; however, a greater range of preclinical models is required to guide its clinical utility in men with mCRPC.

## Figures and Tables

**Table 1:** Currently studied TAAs in prostate cancer CAR-T therapy, and their associated advantages and disadvantages.

Prostate Cancer TAAs	Advantages	Disadvantages
**Prostate-Specific Antigen (PSA)**	Expressed specifically in prostate tissue Stimulates cytotoxic T-lymphocytes in vivo [59]	Strongly expressed in benign prostatic tissue (i.e., benign prostatic hyperplasia)
**Prostate acid phosphatase** **(PAP)**	Secreted by malignant prostate cancer cells Stimulates cytotoxic T-lymphocytes in vivo [31] Clinical success as immunotherapeutic target (Sipuleucel-T) [31]	More highly expressed in well-differentiated cancers (Gleason 6/7) compared to higher grade cancer [52] Expressed in other tissues such as kidneys/testes Secreted in large amounts systemically if prostate is damaged [60] Not expressed on cell surface [51]
**Prostate Stem Cell Antigen** **(PSCA)**	High expression in malignant cancer cells Positive correlation of expression to grade of disease Not released into blood circulation PSCA CAR-T cells have produced promising results in gastric and pancreatic cancers [61,62]	A preclinical study suggested some tumours can ‘escape’ CAR-T cells by means of antigen heterogeneity [63]
**Epithelial cell adhesion molecule (EpCAM)**	Positive correlation of expression to grade of disease EpCAM directed CAR-T therapy in breast cancer has produced promising results [64]	A murine study suggested potential pulmonary toxicity due to EpCAM expression on basal respiratory epithelium [65]
**Prostate-specific membrane antigen (PSMA)**	Positive correlation of expression to grade of disease [66] Trafficking to tumour sites can be imaged [67] High expression related to castration-resistant disease Targets neovasculature involved in metastatic disease [68]	10–15% of prostate cancers do not express PSMA (de-differentiated neuroendocrine variants of prostate cancer express low or absent PSMA) [69,70]

**Table 3:** Preclinical studies, ongoing and completed clinical trials of PSMA-directed CAR-T therapy.

Completed Preclinical Studies						
**Author**	Location		Intervention	Models/Cell Lines Tested		
Maher et al. 2002 [90]	Memorial Sloan-Kettering Cancer Center, USA		2nd-Generation Anti-PSMA CAR-T cells	LNCaP^PSMA +,^ EL4^PSMA+,^ PC-3^PSMA+^ cell lines		
Gade et al. 2005 [91]	Memorial Sloan-Kettering Cancer Center, USA		1st-Generation Anti-PSMA CAR-T cells	LNCaP^PSMA +^ EL4^(PSMA+)^ PC-3^(PSMA+)^ cell lines LNCaP injected mice (orthotopic model) LNCaP/C4-2 injected mice (subcut. model) RM1 injected mice (lung experimental metastasis model)		
Zhong et al. 2010 [94]	Memorial Sloan-Kettering Cancer Center, USA		3rd-Generation Anti-PSMA CAR-T cells	RM1.PGLS^(PSMA+)^ LNCaP^PSMA +,^ EL4^(PSMA+)^ DU145^PSMA−^ cell lines RM1.PGLS^PSMA+^ xenografts		
Zuccolotto et al. 2014 [92]	University of Padua, Italy		2nd-Generation Anti-PSMA CAR-T cells	LNCaP^PSMA +^ PC-3^PSMA+^, PC-3-PIP^PSMA+^ PC-3-PIP injected mice (locoregional + disseminated)		
Ma et al. 2014 [97]	Roger Williams Medical Center, USA		2nd-Generation Anti-PSMA CAR-T cells	PC-3^(PSMA+)^ cell lines PC-3^(PSMA+)^ xenografts		
Santoro et al. 2014 [95]	University of Pennsylvania, USA		3rd-Generation Anti-PSMA CAR-T cells (CD28 + 4-1BB)	MS1^(PSMA+)^, H5V^(PSMA+),^ HMEC-1 ^(PSMA+)^ cell lines + xenografts		
Kloss et al. 2018 [102]	University of Pennsylvania, USA		4th-Generation Anti-PSMA Dominant Negative TGF-beta receptor	PC-3^(PSMA+)^ cell line PC-3^(PSMA+)^ xenografts (metastatic + disseminated)		
Zhang et al. 2018 [100]	Oxford University, United Kingdom		2nd-Generation Anti-PSMA-TGF insensitive CAR-T cells	PC-3 ^PSMA−^, PC-3^(PSMA+)^ LNCaP ^PSMA+^, VCaP ^PSMA+^ cell lines PC-3 ^PSMA−^, PC-3^PSMA+^ xenografts		
Wang et al. 2020 [98]	Shanghai Jiao Tong University, China		IL23mAb- Anti-PSMA-CARs	PC-3^(PSMA+)^ cell line PC-3^(PSMA+)^ xenografts		
Zuccolotto et al. 2020 [96]	University of Padua, Italy		2nd-Generation Anti-PSMA CAR-T cells (CD28) 3rd-Generation Anti-PSMA CAR-T cells (CD28 + 4-1BB)	LNCaP^PSMA +^ PC-3^(PSMA+) cell^ lines		
Hassani et al. 2020 [107]	Tehran University of Medical Sciences, Iran		Anti-PSMA nanobody VHH-CAR-T Cell cells	LNCaP and DU145 cell lines		
Alzubi et al. 2020 [93]	Medical Center, University of Freiburg, Germany		2nd-Generation Anti-PSMA CAR-T cells (4-1BB vs. CD28) + docetaxel	C4-2^PSMA+^, DU145^PSMA^—cell lines C4-2 injected mice (focal model and systemic model)		
Weimin et al. 2020 [101]	First Affiliated Hospital of Xinjiang Medical University, China		2nd-Generation Anti-PSMA CAR-T cells 4th-Generation Anti-PSMA CAR-T inverted receptor (IL7-TGF-beta)	LAPC-9^PSMA+^, LNCaP^PSMA+^ and PC-3^PSMA−^ cell lines LAPC-9 xenografts PSMA expressing PDX		
**Completed Clinical Trials**						
**Identifier**	**Author**	**Type**	**Intervention**	**Enrolment**	**Notable study outcomes**	
NCT00664196 [103] Roger Williams Medical Center, USA	Junghans et al. 2008	Phase I	Nonmyeloablative chemotherapy (CyFlu)—Day −8 Fludarabine—Day (−2 to −6) Anti-PSMA1sr generation CAR-T cells—Day 0 Continuous IV Low dose IL2—for 4 weeks	6 patients with mCRPC	2/5 clinical partial responses (50–70% decline of PSA) No toxicities observed *Study prematurely ended due to funding*	
NCT01140373 [104] (Cohort 1) Memorial Sloan-Kettering Cancer Center, USA	Slovin et al. 2013	Phase I	IV Cyclophosphamide 2nd-Generation Anti-PSMA CAR-T cells Cohort 1—1 × 10^7^ cells/kg; Cohort 2—1.5–3 × 10^7^cells/kg	7 patients with mCRPC	Cohort 1: 1 patient had radiologically stable disease for >6 months; 1 patient had stable disease for >18 months; 2 others had disease progression. Cohort 2: 3 patients developed mild CRS. Cohort 2 ongoing—see below	
**Ongoing Clinical Trials**						
**Identifier**	**Location**	**Type**	**Intervention**	**Measures**	**Enrolment**	**Completion Date**
NCT01140373 [104] (Cohort 2)	Memorial Sloan Kettering Cancer Centre, New York	Phase I	PSMA CAR-T cells + Cyclophosphamide Men with mCRPC	Safety Tolerability Efficacy	13	June 2021
NCT03089203 [105]	University of Pennsylvania,	Phase I	PSMA-specific/TGFβ-resistant CAR modified autologous T cells + Cyclophosphamide Men with mCRPC NCT01140373	Safety Efficacy	18	March 2017
NCT04053062 [108]	Changhai Hospital, Shanghai	Phase I	LIGHT PSMA CAR-T cells + Cyclophosphamide Day 6, Fludarabine Day 4 Men with mCRPC	Toxicity Efficacy	12	July 2021
NCT04249947 [109]	Multiple Centres, United States	Phase I	P-PSMA-101 CAR-T cells following conditioning chemotherapy regimen + Rimiducid Men with mCRPC	Safety Tolerability Efficacy	40	September 2023
NCT04227275 [106]	Multiple Centres, United States	Phase I	CART-PSMA-TGFβRDN cells + cyclophosphamide and fludarabine lymphodepletion Men with mCRPC	Dose limiting Toxicity Safety Efficacy	50	November 2022
NCT04429451 [110]	Multiple Centres, Guangdong, China	Phase I/II	4SCAR-PSMA CAR-T cells Open-label, any PSMA-positive solid tumour	Safety Efficacy Persistence	100	December 2024

Brackets denote the cell line was transduced to express PSMA. CAR, chimeric antigen receptor; CRS: cytokine release syndrome; IL2, interleukin-2; mCRPC, metastatic castrate resistant prostate cancer; PDX, patient-derived xenograft; PSMA, prostate-specific membrane antigen.

**Table 4:** Emerging novel cell surface targets for prostate cancer CAR-T therapy.

Novel Prostate Cancer TAAs	Advantages	Disadvantages
**Immune checkpoint** **B7-H3** **(CD276)**	Elevation of B7-H3 associated with high Gleason score and metastases [112] Also ubiquitously expressed in many cancers Upregulated in prostate cancer stem cells and irradiated prostate tissue with minimal expression in healthy tissue. Enoblituzumab (monoclonal antibody directed at B7-H3 resulted in tumour regression in clinical patients [113] B7-H3 directed CAR-T therapy resulted in tumour lysis/regression in vitro and in vivo, particularly in combination with irradiation [114]	Only one preclinical study has examined B7-H3 targeted CAR-T cell therapy
**Mucin-1** **(MUC-1)**	Positive correlation of expression to grade of disease [115] Has been tested extensively in triple negative breast cancer with some promising results [116] Anti-MUC1 CAR-T cells are able to specifically lyse MUC1 prostate cancer cells in vitro [117]	Expression is heterogeneous, MUC1 expression in prostate cancer ranges between 17–58% in prostate cancer [117,118]
**Interleukin-6 receptor** **(CD126)**	Ubiquitously expressed in a variety of solid tumours CAR-T cells showed potent tumour regression in prostate cancer (DU145) xenograft and multiple myeloma xenograft (RPMI-826) [119].	Lack of research regarding expression patterns in prostate cancer, specifically, as well as on other tissues.
**Lewis-y antigen**	Positive correlation of expression with higher-grade prostate disease, metastatic disease and castrate-resistant disease Promising study demonstrating effective cytokine release and cell lysis as well as tumour burden regression in vivo [120] Ubiquitously expressed in a variety of solid epithelial tumours [121] Promising Phase I results against acute myeloid leukaemia [122]	Only one in vivo study has been conducted specifically on prostate cancer
**STEAP-1** **(six-transmembrane epithelial antigen of prostate type 1)**	Positive correlation of expression with Gleason score A Phase I trial of 77 patients with mCRPC, receiving Anti-STEAP1 antibody supported safety and efficacy [123]	No in vivo or in vitro studies have been conducted specifically against prostate cancer. Expressed in the brain and lungs [124]
